# Association of a *BMP5 *microsatellite with knee osteoarthritis: case-control study

**DOI:** 10.1186/ar4102

**Published:** 2012-11-27

**Authors:** Cristina Rodriguez-Fontenla, Andrew Carr, Juan J Gomez-Reino, Aspasia Tsezou, John Loughlin, Antonio Gonzalez

**Affiliations:** 1 Laboratorio Investigacion 10 & Rheumatology, Instituto de Investigacion Sanitaria - Hospital Clinico Universitario de Santiago, Choupana s/n 15706, Santiago de Compostela, Spain; 2 Nuffield Department of Orthopaedics, Rheumatology and Musculoskeletal Sciences, University of Oxford, Windmill Road, OX3 7HE, Oxford, UK; 3 Department of Medicine, University of Santiago de Compostela, Santiago de Compostela, San Francisco s/n, 15701, Spain; 4 Department of Biology, University of Thessaly, Medical School, 22 Papakyriazi Street, 41222, Larissa, Greece; 5 Institute of Cellular Medicine, Newcastle University, Newcastle upon Tyne NE1 7RU, UK

## Abstract

**Introduction:**

We aimed to explore the involvement of a multiallelic functional polymorphism in knee osteoarthritis (OA) susceptibility as a prototype of possible genetic factors escaping GWAS detection.

**Methods:**

OA patients and controls from three European populations (Greece, Spain and the UK) adding up to 1003 patients (716 women, 287 men) that had undergone total knee joint replacement (TKR) due to severe primary OA and 1543 controls (758 women, 785 men) lacking clinical signs or symptoms of OA were genotyped for the *D6S1276 *microsatellite in intron 1 of *BMP5*. Genotype and mutiallelic trend tests were used to compare cases and controls.

**Results:**

Significant association was found between the microsatellite and knee OA in women (*P *from 3.1 x10^-4 ^to 4.1 x10^-4 ^depending on the test), but not in men. Three of the alleles showed significant differences between patients and controls, one of them of increased risk and two of protection. The gender association and the allele direction of change were very concordant with those previously reported for hip OA.

**Conclusions:**

We have found association of knee OA in women with the *D6S1276 *functional microsatellite that modifies in *cis *the expression of *BMP5 *making this a sounder OA genetic factor and extending its involvement to other joints. This result also shows the interest of analysing other multiallelic polymorphisms.

## Introduction

Investigation of the genetic component of osteoarthritis (OA) susceptibility has yielded the identification of several loci achieving genome-wide significance or consistent replication [1-3]. These loci are insufficient to account for the heritability of OA, which has been estimated as 49 to 78% for knee OA in twin and family studies [4-6]. Multiple possible causes of this discrepancy, which has become known as the missing heritability problem of complex diseases, have been discussed [7]. Some have a particular importance in OA, like the difficulties in discriminating between cases and controls, the variability in phenotype definitions and the relatively small size of genome wide association studies (GWAS) in which OA has been studied in comparison with other diseases [3,8]. Other causes are shared by most complex diseases. They include genetic variants that are not well ascertained with the current GWAS and interactions between genetic variants, and between genetic and environmental factors that are beyond the analysis and design of current studies. Among the variants escaping GWAS detection are rare-frequency variants, even if they have a large effect, and variants with multiple alleles. The latter group is made of large structural variants and small repetitive sequences. They could escape GWAS detection because their multiple alleles cannot have sufficient linkage disequilibrium (LD) with bi-allelic markers as the single nucleotide polymorphisms (SNPs) analyzed in GWAS. One of these multi-allelic polymorphisms, a variable number tandem repeat (VNTR) polymorphism in the gene for asporin, has shown consistent association with OA in Asians and less so in Europeans [9,10]. Other multi-allelic polymorphisms have also been found associated with OA, but none of these studies has been followed by an attempt of replication [11-14].

One of the OA-associated multi-allelic polymorphisms is an intronic microsatellite in *BMP5 *(bone morphogenetic protein 5). It has been associated with hip OA in UK women and its allelic variants showed a regulatory effect on the *BMP5 *promoter *in vitro *[11]. *BMP5 *is a good candidate gene for OA because BMPs are members of the transforming growth factor (TGF)-beta superfamily that were identified by their involvement in cartilage and bone development. Now, they are known to give morphogenetic signals directing tissue organization throughout the body. In particular, *BMP5 *is implicated in bone morphogenesis and in the formation of the skeletal pattern, in addition to having roles in other organs [15]. It is expressed in proliferating zone chondrocytes of the growth plate and is very markedly increased by hypertrophic differentiation. This is in contrast with other BMPs [16]. In chondrocytes, upon binding to the BMP membrane receptors, BMP5 leads to phosphorylation of p38 MAP kinase, ERK and SMAD-1,-5, and -8. Nuclear accumulation of phosphor-SMADs leads to over-expression of genes involved in cartilage homeostasis as proteoglycan and of markers of hypertrophic differentiation [17]. At a cellular level, this translates into an increase of chondrocyte proliferation and in the synthesis of cartilage matrix [16]. In addition, *BMP5 *expression is decreased in OA synovia [18] and bone [19]. These changes in expression together with the *BMP5 *chondrogenic role and involvement in chondrocyte hypertrophy indicate *BMP5 *relevance for OA pathogenesis.

We have genotyped the *D6S1276 BMP5 *microsatellite in a large set of knee OA cases and disease-free controls and found an association with knee OA in women. The associated alleles and the gender specificity are similar to those previously found in UK women with hip OA. This finding reinforces the need to explore the involvement of *BMP5 *genetic variation in OA and extends it to OA in other joints. It also shows that other multi-allelic variants are worthy of examination for their possible involvement in OA.

## Materials and methods

### Patients and controls

OA patients and controls were recruited at Thessaly in Greece, Santiago de Compostela in Spain and Oxford in the UK. All were European Caucasians. There were 1,003 patients (716 women, 287 men) who had undergone total knee joint replacement (TKR) due to severe primary OA. The 1,543 controls (758 women, 785 men) lacked clinical signs of OA. Full details about the patients and the controls have been reported [20]. In the Greek collection, there were 369 TKR patients (298 women and 71 men) and 383 controls (241 women and 142 men). TKR patients had a Kellgren-Lawrence (K/L) grade > 2 prior to surgery and an age range of 40 to 90 years. Rheumatoid arthritis, other autoimmune diseases, chondrodysplasias, infection-induced OA, and post-traumatic OA were excluded. The controls had a K/L grade of 0 and had undergone treatment for injuries and fractures. They were from 46 to 88 years old. In the Spanish collection, there were 274 TKR patients (222 women and 52 men) and 462 controls (161 women and 301 men). Patients were selected as consecutive patients, of 55-80 years of age at the time of surgery, and undergoing TKR if a rheumatologist considered them to suffer from primary OA. Exclusion criteria were inflammatory, infectious, or traumatic joint pathology and lesions due to crystal deposition or osteonecrosis. Controls were older than 55 years of age and were selected during preoperative work-up for elective surgery other than joint surgery and without symptoms or signs of OA. The UK collection included 360 TKR patients (196 women and 164 men) of 56 to 85 years of age and 698 controls (356 women and 342 men) of 55 to 89 years of age. Patients showed symptoms and signs of OA of sufficient severity to require surgery, including a K/L grade ≥ 2 (> 90% of them had grade 3 or 4). Inflammatory arthritis (rheumatoid, polyarthritic or autoimmune disease) was excluded, as was post-traumatic or post-septic arthritis. Controls had no signs or symptoms of arthritis or joint disease (pain, swelling, tenderness or restriction of movement). All patients and controls gave their written informed consent and the use of their DNA for OA genetic studies was approved by the respective ethics committees.

### Genotyping

The BMP5 microsatellite *D6S1276 *was genotyped by length analysis of the PCR products obtained with primers 5'-FAM-atgcctggcaaatgtcaagt-3' and 5'-gcccagcatccctgattaag-3'. Size of the fluorescence-labeled products was determined by capillary electrophoresis on an ABI 3130xl Avant Genetic Analyzer (Applied Biosystems, Foster City, CA, USA) and the corresponding microsatellite genotypes were determined using GeneMapper 3.5 software (Applied Biosystems). Several samples with different genotypes were sequenced to assess the accuracy of results with the Big Dye v3.1 Ready Reaction Kit (Applied Biosystems).

### Statistical analysis

Analyses were based on Power Marker software [21] and on the R software implementation of the Cochran-Mantel-Haenszel stratified test [22]. Exact tests for Hardy Weinberg equilibrium (HWE) were run with the Markov Chain Monte Carlo method. Deviation from HWE motivated analyses based in genotype 2 × n contingency tables and the multi-allelic trend test. These two tests are totally insensitive to HWE deviations [23]. Significance of the genotype test was obtained with the Markov Chain Monte Carlo method to avoid the limitations that sparse contingency tables pose to the asymptotic chi squared distribution method. Rare genotypes, with frequency below 1.0% were excluded from analysis. In this way, the degrees of freedom were reduced and the tests became more robust. These analyses were conducted collection by collection and with the three collections together. Heterogeneity between collections was assessed with the population differentiation test applied to the controls and with the inconsistency (*I*^2^) statistic [24] applied to the Cramer's V effect size, which is the effect size most commonly used for 2 × n contingency tables. Combined analysis was done considering all data together and stratified by sample collection with the Cochran-Mantel-Haenszel method for 2 × n tables.

## Results

### *D6S1276 *genotypes

A total of 2,545 DNA samples were available for study. Valid genotypes for the *D6S1276 *microsatellite were obtained from 93.32% of them with a similar distribution of the genotype call rate between the three sample collections and between cases and controls. A total of ten different alleles were observed, ranging from five to fourteen tetranucleotide repeats (Table 1). The most common was the ten-repeats allele (41.2%), followed by the eight-, nine- and eleven-repeats alleles (20.1, 20.2% and 13.4%, respectively). All the remaining alleles showed a frequency below 1.0%. These 10 alleles appeared in 28 different genotypes (not shown). The most frequent was the 10/10 genotype (17.7%) followed by the 10/9 and 10/8 genotypes (16.8 and 15.9%, respectively). Up to 14 genotypes showed a frequency below 1.0% and they were excluded from further study (for remaining genotype frequencies, see Tables S1- S3 in Additional file 1). In this way, 62 samples (or 2.4% of all the genotyped samples) were left out, with 2,313 remaining in subsequent analyses. They showed 14 different genotypes made of five alleles (from seven to eleven tetranucletotide repeats).

**Table 1:** Allele counts and frequency of the *D6S1276 *BMP5 microsatellite in patients with knee osteoarthritis (OA) and in controls from the three sample collections

	Controls								Patients with knee OA							
Allele	Spain		Greece		UK		Total		Spain		Greece		UK		Total	
	Count	%	Count	%	Count	%	Count	%	Count	%	Count	%	Count	%	Count	%
5	1	0.1					1	0.0					3	0.4	3	0.2
6	1	0.1	2	0.3	2	0.2	5	0.2	1	0.2	4	0.6			5	0.3
7	39	4.4	23	3.4	58	4.4	120	4.2	18	3.6	12	1.7	35	4.9	65	3.4
8	160	18.2	126	18.9	256	19.6	542	19.0	116	23.2	139	20.1	158	22.3	413	21.7
9	188	21.4	147	22.0	282	21.6	617	21.6	97	19.4	139	20.1	104	14.7	340	17.9
10	362	41.2	264	39.5	525	40.3	1151	40.4	210	42.0	292	42.2	303	42.8	805	42.4
11	115	13.1	98	14.7	168	12.9	381	13.4	53	10.6	102	14.7	101	14.3	256	13.5
12	11	1.3	6	0.9	12	0.9	29	1.0	5	1.0	4	0.6	4	0.6	13	0.7
13			2	0.3	1	0.1	3	0.1								
14	1	0.1					1	0.0								

Total	878		668		1,304		2,850		500		692		708		1,900	

There was a significant deviation from HWE in the UK (*P *= 0.008) controls, but not in the Spanish (*P *= 0.6) or Greek controls (*P *= 0.3). The deviation motivated a careful revision of the genotypes, and their confirmation by sequencing. Also we compared the reported BMP5 allele frequency distribution [11] with the one obtained by us in UK women and no difference was found (*P *= 0.7). In addition, no difference was detected between the three collections in our study (*P *= 0.9). These results showed the accuracy of the genotypes. However, we used tests insensitive to deviations from HWE as more appropriate for our data: genotype test and the multi-allelic trend tests [23].

### Association analyses by sample collection

Previously reported association of *BMP5 *with hip OA was only observed in women [11], therefore a gender-stratified analysis was done (Tables 2 and 3). Significant differences between knee OA patients and controls were only observed in women from the UK and from Greece (Table 4). In the Greek women, the multi-allelic trend test and the genotype test provided very similar results, with a significant difference between knee OA patients and controls in both of them (*P *= 0.021 and *P *= 0.032). In the UK women the two tests were discordant. The multi-allelic trend test showed a significant difference (*P *= 0.028), whereas the genotype test was not significant (*P *= 0.3). This discordance could be partly related to the greater power of the first test that has four degrees of freedom, in contrast with the thirteen corresponding to the genotype test. On comparison, no significant differences were observed in the Spanish women or in any of the men (Table 4).

**Table 2:** Allele counts and frequency of the *D6S1276 *BMP5 microsatellite in women with knee osteoarthritis (OA) and control women from the three sample collections

	Controls								Patients with knee OA							
	Spain		Greece		UK		Total		Spain		Greece		UK		Total	
Alleles	Count	%	Count	%	Count	%	Count	%	Count	%	Count	%	Count	%	Count	%
5													1	0.3	1	0.1
6					1	0.2	1	0.1	1	0.2	4	0.7			5	0.4
7	16	5.2	20	4.8	28	4.4	64	4.7	16	3.9	11	2	17	4.4	44	3.2
8	52	17	70	16.8	117	18.2	239	17.5	91	22.2	114	20.2	95	24.6	300	22.1
9	66	21.6	99	23.8	137	21.3	302	22.1	82	20	113	20	56	14.5	251	18.5
10	126	41.2	159	38.2	263	41	548	40.2	174	42.4	240	42.6	164	42.5	578	42.5
11	42	13.7	65	15.6	87	13.6	194	14.2	43	10.5	79	14	50	13	172	12.6
12	4	1.3	3	0.7	9	1.4	16	1.2	3	0.7	3	0.5	3	0.8	9	0.7

Total	306		416		642		1,364		410		564		386		1,360	

**Table 3:** Allele counts and frequency of the *D6S1276 *BMP5 microsatellite in men with knee osteoarthritis (OA) and in control men from the three sample collections

	Controls								Knee OA							
	Spain		Greece		UK		Total		Spain		Greece		UK		Total	
Alleles	Count	%	Count	%	Count	%	Count	%	Count	%	Count	%	Count	%	Count	%
5	1	0.2					1	0.1					2	0.6	2	0.4
6	1	0.2	2	0.8	1	0.2	4	0.3								
7	23	4	3	1.2	30	4.5	56	3.8	2	2.2	1	0.8	18	5.6	21	3.9
8	108	18.9	56	22.2	139	21	303	20.4	25	27.8	25	19.5	63	19.5	113	20.9
9	122	21.3	48	19	145	21.9	315	21.2	15	16.7	26	20.3	48	14.9	89	16.5
10	236	41.3	105	41.7	262	39.6	603	40.6	36	40	52	40.6	139	43.2	227	42.0
11	73	12.8	33	13.1	81	12.2	187	12.6	10	11.1	23	18	51	15.8	84	15.6
12	7	1.2	3	1.2	3	0.5	13	0.9	2	2.2	1	0.8	1	0.3	4	0.7
13			2	0.8	1	0.2	3	0.2								
14	1	0.2					1	0.1								

Total	572		252		662		1,486		90		128		322		540	

**Table 4:** Association of the *D6S1276 *BMP5 microsatellite with knee osteoarthritis (OA) by sample collection and by gender

		Number		*P-*values	
Collection	Gender	Controls	Knee OA	Multi-allelic trend test	Genotype test
Spain	women	149	200	0.2	0.5
	men	277	43	0.4	0.4
	all	426	243	0.1	0.5
Greece	women	205	275	0.021	0.032
	men	120	63	0.8	0.6
	all	325	338	0.2	0.09
UK	women	312	187	0.028	0.2
	men	325	157	0.09	0.3
	all	637	344	0.0059	0.1

### Combined association across sample collections

In a first analysis, we simply combined all samples together without considering their origin (Table 5). This approach was justified in the lack of heterogeneity of *D6S1276 *effects between the three sample collections (*I*^2 ^= 0.0%) and lack of differences in the microsatellite genotypes between the three populations (above). Both the multi-allelic trend test and the genotype test were clearly significant in women (*P *= 3.3 × 10^-4^, and *P *= 4.1 × 10^-4^, respectively). We also performed a combined analysis accounting for origin of the samples with the Cochran-Mantel-Haenszel test applied to the 2 × 14 × 3 genotype contingency table (Table 5). The result was very similar to the simple pooling showing association in the female patients (*P *= 3.8 × 10^-4^). No association was observed in men (Table 3).

**Table 5:** Association of the *D6S1276 *BMP5 microsatellite with knee osteoarthritis (OA0 across sample collections

	Multi-allelic trend test	Genotype test	
Gender	Simple pooling	Simple pooling	CMH^a^
Women	0.00033	0.00041	0.00038
Men	0.11	0.2	0.4
All	0.00073	0.0076	0.0092

^a^CMH, Cochran-Mantel-Haenszel stratified analysis

**Figure 1:**
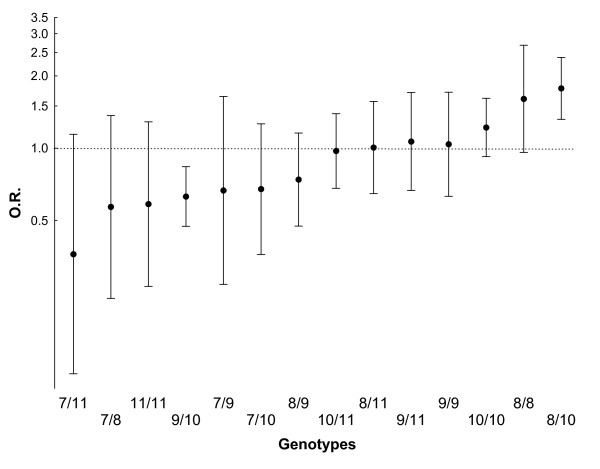
**Comparison of the individual genotypes between women with knee osteoarthritis (OA) and controls**. The odds ratio (OR) and 95% CI corresponding to the specified genotypes relative to the remaining are represented for frequencies in patients in relation to controls. The order is from protective to risk genotype.

Inspection of the genotypes showed that the difference between female patients with knee OA and female controls was not due to a single genotype but to several (Figure 1), including an increase in patients of the 10/8 genotype (20.4% vs. 12.6%, odds ratio (OR) 1.77, 95% CI 1.32-2.39, *P *= 0.0002) and a decrease of the 10/9 genotype (14.4% vs. 21.0%, OR 0.63, 95% CI 0.47-0.84, *P *= 0.002). No differences were detected when the same comparisons were made in men although variation was in the same direction

**Figure 2:**
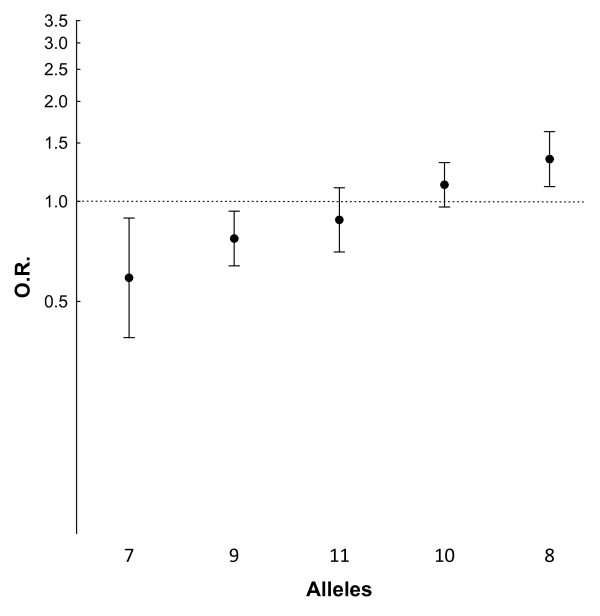
**Comparison of the individual alleles between women with knee osteoarthritis (OA) and controls**. The odds ratio (OR) and 95% CI corresponding to the specified alleles relative to the remaining are represented for frequencies in patients in relation to controls. The order is from protective to risk allele.

## Discussion

Our study has identified the *D6S1276 *microsatellite in *BMP5 *as a new genetic factor for knee OA in women. The *D6S1276 *microsatellite has previously been associated with hip OA [11]. Concordance with this study extends beyond the association because the two studies found association in women and with a similar pattern of alleles. The concordance of our findings was very clear with the first set of samples in the hip OA study, where the risk allele was also the eight-repeats allele (*P *= 0.006) and protection was mediated by the seven-repeats allele (*P *= 0.03), and other alleles showed changes in the same direction [11]. The second set of samples was not associated with *D6S1276 *in Wilkins *et al*., but in the combined analysis of the two sets the association remained as well as the protective effect of the seven-repeats allele (*P *= 0.03) and the direction of change of the eight-repeats allele. Therefore, our study does not amount to a formal replication because of the difference in the involved joint, but strongly reinforces the evidence in support of the implication of this *BMP5 *polymorphism in OA susceptibility. In addition, our finding indicates that this microsatellite could be associated with independence of the joint.

It is very unlikely that the association of *D6S1276 *with knee OA could be due to confounding by population stratification. The association was found in two independent sample collections with origin in Greece and the UK, and reinforced in the analyses with samples from Spain without any detectable heterogeneity as shown by the lack of inconsistency (*I*^2 ^= 0%) and by the very similar results obtained pooling data from the three collections or using the Cochran-Mantel-Haenszel approach. Lack of differences in *D6S1276 *between collections was also shown by the absence of population differentiation between the controls (*P *= 0.91), and by lack of significant differences in any of the pairwise comparisons between controls, and differences only in patients from Greece (Table S2 in Additional file 1). In addition, there are arguments to exclude significant population stratification in each of the three sample collections taken individually. The UK samples were genotyped in the recently reported arcOGEN GWAS [1]. In this study no significant population stratification was detected (genomic control inflation factor λ_1000 _= 1.009). The Spanish samples are from subjects reporting a Spanish origin extending for two or more generations. In fact, 94.7% of the patients and 95.4% of the controls are of an even more homogeneous ancestry, as all their known ancestors were from Galicia, a region in the North-West of Spain. Excluding subjects of a wider Spanish ancestry did not alter the results (not shown). Finally, the Greek collection was made of individuals of Greek descent who were inhabitants of Central Greece and ethnic or racial differences between patients and controls were not detected in multiple previous studies [13,14,20,25].

The identification of the *D6S1276 *microsatellite as an important polymorphism in hip OA has been the fruit of successive studies by the group of John Loughlin [11,26-29]. Initially, linkage with hip OA was detected in chromosome 6 after stratification by affected joint of a genome-wide linkage study performed in sibling-pairs who had undergone total hip replacement (THR) for primary OA [26]. The linked region was very broad (> 50 cM). Fine mapping with additional microsatellites and THR families reduce the locus to 11.4 cM at 6p12.3-q13 and showed that it was specific to women [27]. This study also increased the statistical evidence for linkage. Two candidate genes in the interval were excluded by association analysis with SNPs mapping to their coding sequence and promoter, *COL9A1 *[27] and *BMP5 *[28]. However, a subsequent study including additional microsatellites confirmed the same peak of linkage and found association in intron 1 (*D6S1276*) and 3' to *BMP5*, bringing forward the possibility that the causal polymorphism could be in *cis*-regulatory sequences of this gene [29]. This hypothesis was tested in the last study to date, where more samples and polymorphisms within intron 1 were analyzed [11]. Here, association was confirmed with *D6S1276 *and found only with one of the new polymorphisms. Subsequent functional analysis with reporter gene assays showed that only *D6S1276 *was able to modify the expression of the luciferase gene under the *BMP5 *promoter [11]. This analysis also demonstrated a significant amount of variability of modifier effect depending on the cell line where the study was done, the *D6S1276 *allele and the alleles at three nearby SNPs.

The variability of effects of *D6S1276 *is congruent with what is known of the regulation of BMP5. In effect, regulation of this gene is very modular, precise and complex involving multiple *cis*-acting enhancers with tissue- and location-specific effects [17,30,31]. This has been clearly demonstrated in the mouse, where mutations in BMP-5 showed cartilage and bone defects affecting seemingly unrelated skeletal elements in addition to several soft-tissue organs [15]. This indicated a complex pattern of transcription regulation that was confirmed with the identification of multiple *cis*-regulatory modular enhancers each of them acting in particular tissues or even in different regions of the same organ [30,31]. Most of these enhancers are 3' to the coding sequence but also an enhancer directing expression in different areas of the mouse ribs has been identified in one of the *Bmp5 *introns [31]. A similar complex and precise regulation seems to be present in humans because analysis of differential allelic expression showed extreme variability between the joint tissues with differences even between regions of the same cartilage surface in function of their proximity to the OA lesion [32].

Association of *D6S1276 *is not contradicted by the lack of replication in the Spanish collection because it was most likely due to lack of power (below 0.46 for the most significant differences found in the current study) and not to significant differences between the Spanish and the other two collections (Table S4 in Additional file 1). In addition, the association we have found was not questioned by the lack of an association signal in the GWAS because the multiplicity of the microsatellite-associated alleles makes for very poor correlation with any bi-allelic SNP. This has been already shown in the previous studies of hip OA, where none of the *BMP5 *SNPs analyzed accounted for the association of *D6S1276 *[11,28]. Therefore, our initial hypothesis positing that some of the unidentified genetic susceptibility to OA could be in multi-allelic polymorphisms is supported by our results in *BMP5 *and should motivate analysis of other similar loci. In addition, the lack of linkage with knee OA in the original genome-wide linkage study that brought attention to the *BMP5 *locus is not against the association we have found, because this study included fewer families with knee OA (34 families, including affected women and men) than with hip OA in women (85 families plus 44 families with hip OA in men), and therefore was less sensitive to detect any difference specific to the women of this subgroup [26].

## Conclusions

We have found association of knee OA in women with the *D6S1276 *functional microsatellite that modifies in *cis *the expression of *BMP5*. The results are very concordant with those observed previously for hip OA, making this association sounder, and extending its range of involvement to other joints. These results also show the interest of analyzing other multi-allelic polymorphisms for their possible role in OA susceptibility.

## Abbreviations

BMP5: bone morphogenetic protein 5; HWE: Hardy Weinberg equilibrium; K/L: Kellgren-Lawrence; LD. linkage disequilibrium; OA: osteoarthritis; PCR: polymerase chain reaction; SNP: single nucleotide polymorphism; THR: total hip replacement; TKR: total knee replacement; VNTR: variable number tandem repeat.

## Competing interests

The authors declare that they have no competing interests.

## Authors' contributions

C R-F contributed to the acquisition and analysis of data, drafting the article and final approval of the submitted version. AC, JJG-R, AT and JL contributed to the acquisition and interpretation of data, critical revision of the article for important intellectual content and final approval of the submitted version. AG contributed to the conception and design of the study, analysis and interpretation of data, drafting the article and final approval of the submitted version. AG takes responsibility for the integrity of the work as a whole, from inception to finished article. All authors read and approved the final manuscript.

## Supplementary Material

Additional file 1 **Genotype frequencies and comparison across populations**. Genotype counts and frequencies in patients with knee OA and in controls from the three sample collections are provided as total, in women and in men. The genotype frequencies were also compared pairwise between the sample collections. Click here for file
